# Highly sensitive image-derived indices of water-stressed plants using hyperspectral imaging in SWIR and histogram analysis

**DOI:** 10.1038/srep15919

**Published:** 2015-11-04

**Authors:** David M. Kim, Hairong Zhang, Haiying Zhou, Tommy Du, Qian Wu, Todd C. Mockler, Mikhail Y. Berezin

**Affiliations:** 1 Department of Radiology, Washington University School of Medicine, St. Louis, MO 63110; 2 Donald Danforth Plant Science Center, St. Louis, MO 63132.

## Abstract

The optical signature of leaves is an important monitoring and predictive parameter for a variety of biotic and abiotic stresses, including drought. Such signatures derived from spectroscopic measurements provide vegetation indices – a quantitative method for assessing plant health. However, the commonly used metrics suffer from low sensitivity. Relatively small changes in water content in moderately stressed plants demand high-contrast imaging to distinguish affected plants. We present a new approach in deriving sensitive indices using hyperspectral imaging in a short-wave infrared range from 800 nm to 1600 nm. Our method, based on high spectral resolution (1.56 nm) instrumentation and image processing algorithms (quantitative histogram analysis), enables us to distinguish a moderate water stress equivalent of 20% relative water content (RWC). The identified image-derived indices 15XX nm/14XX nm (i.e. 1529 nm/1416 nm) were superior to common vegetation indices, such as WBI, MSI, and NDWI, with significantly better sensitivity, enabling early diagnostics of plant health.

The current fundamental goal in monitoring vegetation is to identify physical and chemical changes in plants in response to environmental stress and gene alteration with high sensitivity^1^,^2^,^3^. Determining quantitative relationships between optical characteristics and plant chemical composition, such as water content, requires the use of spectral signatures. These signatures are traditionally derived using spectroscopy, where part of the plant, usually a leaf, is analyzed for spectral alteration^4^. A typical setup features an integrating sphere, a reasonably high resolution (<1 nm) spectrometer, and a set of detectors sensitive from 350 to 2500 nm^5^,^6^,^7^. Recorded spectra are then processed to establish a correlation between the spectral features and chemical composition, thus providing plant indices of stress^8^,^9^.

This spectroscopy approach has several shortcomings, such as weak correlation between the suggested index and the parameter of interest, and low sensitivity that prevents identification of even moderate stress. In part, these shortcomings are due to inherent limitations of the spectroscopy-derived indices that are calculated as an average across a large area and ignore spectral variance within the sample^10^. Hence, identification of more reliable techniques with better sensitivity for early assessment of plant stress has been recognized as a major challenge in plant research^3^.

Hyperspectral imaging (HSI), where high spectral resolution is combined with spatial resolution, minimizes the averaging of spatial information. In this approach, every pixel on a 2D image carries a complete spectrum; altogether the image is a 3D dataset (datacube). The method is similar to spectroscopy, but requires special hardware, such as pixel-by-pixel scanners, push-broom imaging spectrographs, or specialized 2D cameras with sensor-integrated color filters.

While large-scale hyperspectral approaches have been broadly accepted in remote sensing of vegetation over the last 20 years^11^,^12^,^13^, HSI has only recently found its way into a small scale controlled environments^14^,^15^,^16^. This small scale helps to identify the spectral signatures of the vegetation under controlled laboratory conditions and then apply the recovered indices to the field studies. In addition to the cost-effectiveness of this approach, this strategy promises to maximize the sensitivity of the hyperspectral methods and take into account a variety of environmental factors.

Current HSI in small scale plant research is focused on chlorophylls and other plant-related endogenous pigments, as well as on chromophores optically active in the visible spectral range (400–800 nm)^17^,^18^. Activity in this research area has been primarily due to the accessibility of silicon-based detectors (2D CCD and CMOS cameras, photomultiplier tubes (PMTs)) with sufficient sensitivity up to 1000 nm and the availability of the corresponding optics. Other noticeable endogenous chromophores in plants, such as water, lipids, nitrogen, and starch, require imaging techniques in the short-wave infrared (SWIR) range (900–2,500 nm). Measurements in SWIR necessitate a different set of detectors (often several detectors together) and optical elements that until recently were not commercially available or optimized for imaging. Hence, there are limited HSI data and almost no imaging studies connecting chemical composition to optical signature in SWIR on a small scale. To the best of our knowledge, only one study highlighting the potential of HSI-SWIR in plant research has been described, with no image analysis provided^19^.

To address the importance of effective monitoring of plant nutrition and early diagnostics of plant stress, we developed a 1.56 nm resolution HSI-SWIR system and image-guided algorithms. With this system, we revisited known band-based indices of water-deficient plants and derived sensitive image-based metrics for plants experiencing moderate water stress.

## Results and Discussion

### Relative water content (RWC) identifies models of moderate stress in boxwood plants

**Figure 1:**
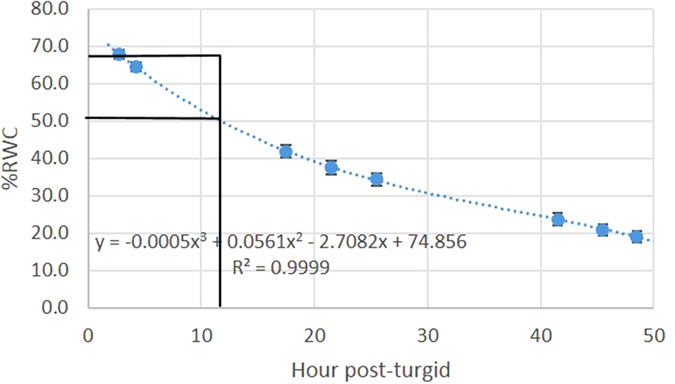
RWC of boxwood leaves at different post-turgid time points. 2 h and 12 h post-detachment leaves correspond to an approximately 20 ± 2.4% difference in RWC levels. A trend-line (dotted) reflects polynomial decay (3-rd order) (R^2^ = 0.9999). Solid lines: RWC values at 2 h and 12 hours. Error bars correspond to standard error, n = 7 leaves.

Typical values of relative water content (RWC)^20^ in leaves range between 98% in turgid (saturated with water) and transpiring leaves to ~20–50%, a level that is equivalent to a moderate-to-strong stress^21^,^22^, with the lost water content of 70% found in severely desiccated plants that cannot be recovered. To identify a model for moderate stress (equivalent to the loss of ca. 20% water in many species^23^) we used naturally desiccated boxwood leaves. The RWC values of leaves detached from the plant at different time points (2–50 hours post-detachment) served as a guide for stress level. Interpolation of the experimentally measured RWC data (Fig. 1) suggested that 12 h post-turgid leaves have a moisture content approximately 20 ± 2.4% lower than 2 h post-turgid leaves.

**Figure 2:**
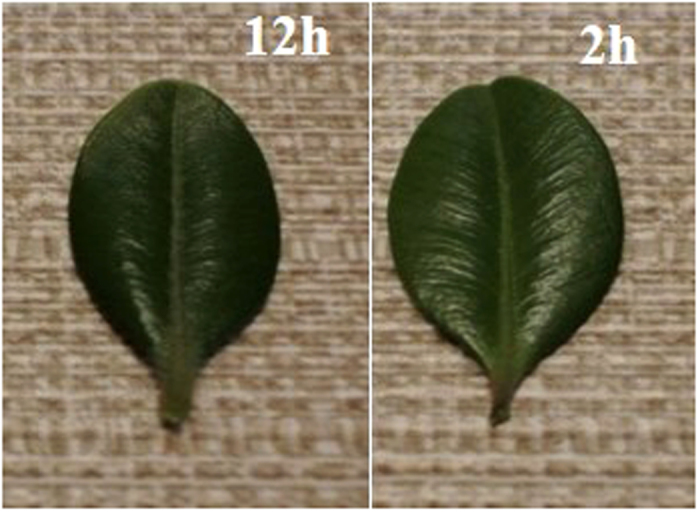
Images of leaves. Reflection image of the leaves taken with a color camera: Nikon 1J, objective 1 NIKKOR, 18.5 mm f/1.8, RGB.

Visual observation of 2 h and 12 h leaves using a conventional digital color camera showed apparent features of water stress: mild wilting and darker green color (Fig. 2). The former is due to the decrease in cellular turgor pressure that affects the shape of the leaf^24^. The latter is attributed to the difference in chlorophyll reflection, where greener (darker) leaves demonstrate lower reflection. However, it was difficult to visually distinguish between the two types of leaves with confidence and a different method was needed and developed in this study.

### RWC values and spectral parameters in SWIR are correlated

Since water absorbs strongly in SWIR, the optical properties of leaves are expected to correlate with RWC values^25^. As measured in the integrating sphere (see the design in Supplementary Fig. 1) and in accordance with literature data^26^, boxwood leaves show characteristic water-related bands at 975, 1200, and 1470 nm, with relatively low variability in their reflectance and transmission SWIR spectra (Supplementary Fig. 2).

**Figure 3:**
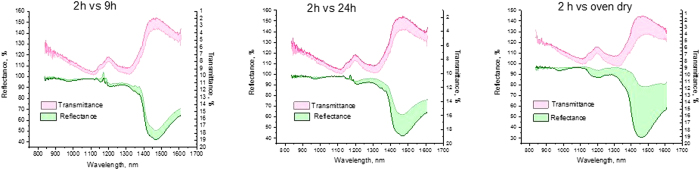
Change in transmission and reflectance spectra of leaves upon drying at different times post-detachment. Shaded area indicates the change from relatively fresh (2 h) to desiccated leaves (naturally dried for 9 h, 24 h, and oven dry).

The decreases in intensity of the water-specific bands (illustrated in Fig. 3) upon drying were correlated with the loss of moisture content. The absorption values and their changes calculated from the reflection and transmission measurements (Supplementary Figs. 3–4) were also correlated with the RWC values in accordance with the Lambert-Beer law, confirming the loss of ca. 20% of water in the 12 h leaves compared to the 2 h ones. Since such loss corresponds to moderate stress, the two types of leaves were used as models for further HIS-SWIR studies.

### Quantitative histogram image analysis in SWIR

Three-dimensional SWIR datasets (SWIR datacubes) were collected using a home-built imager described in Supplementary Fig. 5 and analyzed by the in-house developed software. The datacube presents a large number of opportunities for generating a variety of images (i.e. images based on individual bands, bands ratio, principle components, etc.), with the challenge of identifying the image with the best (highest) contrast.

We started the data analysis from the broadband images. These broadband (800–1600 nm) SWIR images of leaves revealed some signal heterogeneity within each leaf (lamina vs midrib, edges vs lamina, etc). Such heterogeneity reflected non-uniform chemical composition within the leaf (due mostly to the water gradient across the leaf), as well as differences in the leaf’s thickness in accordance with the radiative transfer model of plant leaf transmittance^10^.

**Figure 4:**
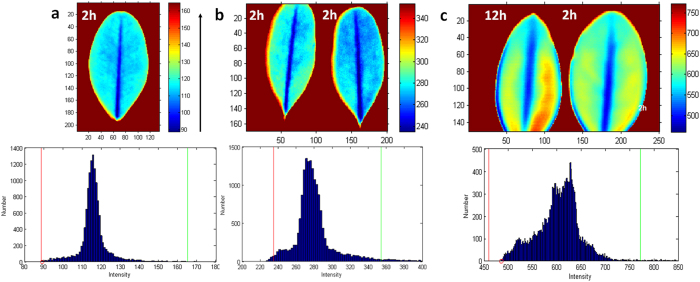
Broadband SWIR images of boxwood leaves and corresponding histograms. (**a**) Single leaf (2 h), (**b**) leaves with similar moisture levels (2 h post-detachment), (**c**) leaves with a 20% difference in moisture level (2 h and 12 h post-detachment). All images were optimized for the best visual appearance by adjusting the corresponding image histogram. Colormap “jet”: arrow shows the increase in transmission intensity. Images intensities were normalized to the light source.

To quantify the contrast in the images we used image histogram analysis. The histogram of an image (such as shown in Fig. 4) refers to a plot of pixel intensity values, where the X axis corresponds to the intensity and the Y axis to the number of pixels at each unique intensity value found in that image.

A single leaf presented a mono-modal Gaussian-type histogram (Fig. 4a), reflecting signal heterogeneity within the leaf. A histogram with two leaves detached at the same time (2 h), and therefore having a comparable level of water content, showed a deviation from the Gaussian shape characteristic of the mixture from two samples (Fig. 4b). Leaves with different moisture contents (such as 2 h and 12 h post-detachment) predictably revealed a more complex histogram (Fig. 4c).

We envisioned that bimodal distribution of the histogram, where the pixel intensities are clustered around two reasonably-separated values, might serve as a benchmark for contrast evaluation and provide a new approach for finding the best index with the highest sensitivity. Highly overlapped modes (i.e. monomodal distributions) suggest a low contrast with weak to no difference between the leaves, while well separated modes would indicate a high contrast.

A bimodality test of the image histogram can be performed by calculating a parameter that takes into account the width and depth of the valley separating the histogram modes. In the Bhattacharyya method^27^, separation between the two modes is achieved by fitting the histogram intensity to the bimodal Gaussian distribution and calculation of the Bhattacharyya distance (*D*_*B*_) or its closely related Bhattacharyya coefficient (*C*_*B*_) from the means of the peaks and their standard deviations (see an example shown in Supplementary Fig. 6). While *D*_*B*_ provides the separation between the distinct populations, *C*_*B*_ measures overlap between two populations. The two bimodality parameters are related (See Methods, eq. 9): *D*_*B*_ is the preferable parameter to quantify image histograms with reasonably separated peaks, while *C*_*B*_ is more informative for images with highly overlapped modes. The range for *D*_*B*_ is from 0 (no separation between the modes, identical populations) to infinity (large separation, no overlap). The values for *C*_*B*_ lie between 0 (no overlap) to 1 (complete overlap, identical populations). From analysis of plant images in this work, we suggest that images with *D*_*B*_ < 0.6 correspond to poor contrast where noise prevails, while images with *D*_*B*_ > 1.0 represent moderate, and *D*_*B*_ > 3.0 indicate strong contrast. In the following results all indices were compared using a bimodality test with Bhattacharya distance.

Based on these quantitative metrics, images of leaves acquired in the broadband range 800–1600 nm showed poor contrast (Fig. 4c) with *D*_*B*_ = 0.286 for plants with a different level of moisture (all contrast parameters are tabulated in Table 1).

### Single band images show low contrast

To improve the contrast, we investigated narrowband images with spectral bands of 1.56 nm. Noticeable differences between the 2 h and 12 h leaves were seen in the 1300–1500 nm range (Supplementary Fig. 7, see also a full set of images recorded at all wavelengths from 800 to 1600 nm with 1.56 nm increments, for a total 512 images, as a movie file in Supplementary Movie). Since the largest change in the spectra of drying leaves occurs at ca. 1470 nm, higher sensitivity was expected in this spectral band. The bimodality level at 1470 nm (*D*_*B*_ = 0.536) was, however, below the minimally acceptable contrast of *D*_*B*_ < 0.6. Leaves with the same level of moisture (detached from the plant at the same time) predictably showed a similar low contrast (see Supplementary Figs. 8–9) with the *D*_*B*_ = 0.565 at 1470 nm. Comparable *D*_*B*_ values were apparently due to spatial variability within the leaf (single leaf showed *D*_*B*_ = 0.530 at 1470 nm), rather than the difference between the leaves. Thus, the sensitivity of single-band imaging to identify stress with sufficient contrast was low, precluding this method from practical use.

### Band ratio increases contrast and sensitivity

**Figure 5:**
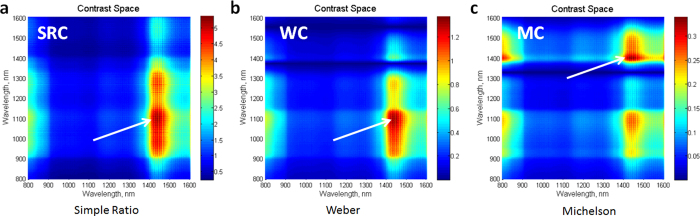
Calculated contrast space in hyperspectral images. (**a**) Simple Contrast ratio (SRC), (**b**) Weber contrast (WC) and (**c**) Michelson contrast (MC). Each contrast space shows 262,144 contrast values. Arrows point to areas with the highest intensity values. Red color corresponds to higher intensity values. X-axis is the monitoring wavelength and y-axis the normalizing wavelength.

To increase sensitivity, we used the ratio of band intensity, rather than that of individual bands. A similar approach has long been used in remote sensing of vegetation^13^. An exhaustive search across the datacube for the highest visual contrasts was initially conducted using established metrics, such as a simple ratio contrast (SRC), Weber contrast (WC)^28^, and Michelson contrast (MC)^29^,^30^. Contrast values (total number 512 × 512 = 262,144) were plotted against two wavebands in a 3D plot (Fig. 5), where the red areas indicate images with the highest contrast. The resulting plot yielded a contrast space to identify the cluster(s) of images with the highest intensity ratios.

**Figure 6:**
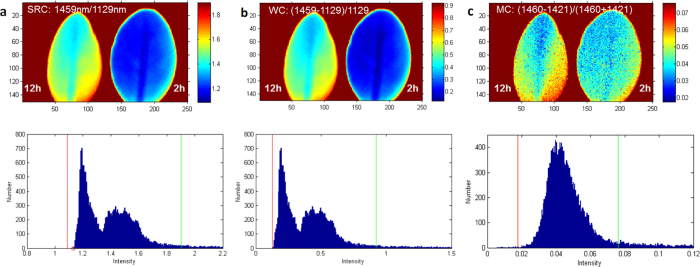
Images of leaves using band ratios from SRC, WC and MC (**a**) using the highest values from SRC, (**b**) using the highest values from WB, (**c**) using the highest values from MC. The images are shown within given boundaries on the corresponding histograms. Blue pixels correspond to the histogram with lower intensity, red to higher intensity.

Both SRC (1459 nm/1129 nm) and WC ((1459 nm–1129 nm)/1129 nm) provided satisfactory contrast between the leaves with *D*_*B*_ = 1.142 (Fig. 6a,b). The MC (1460 nm–1421 nm)/ (1460 nm + 1421 nm) metric showed almost no contrast (Fig. 6c) with a low *D*_*B*_ = 0.239. Despite the promising results from SRC and WC with *D*_*B*_ > 1.0, we continued to investigate the datacube to identify images with higher contrast.

### Novel band ratio reveals the highest visual contrast

The search for better contrast continued by visualizing band ratio images in a movie format and selecting the frames with the best visual contrast. The images with satisfactory visual contrast were then tested for bimodality.

**Figure 7:**
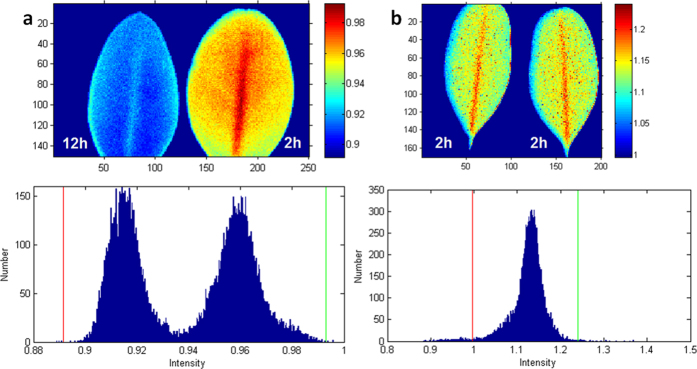
Images at 1529 nm divided over 1416 nm. (**a**) Leaves with different moisture levels (2 h and 12 h leaves). (**b**) Leaves with the similar moisture levels (both 2 h leaves). All images are shown for pixels within given boundaries on the corresponding histogram.

This non-exhaustive screening revealed that images recorded within the range 1500–1590 nm divided over images recorded at the range 1390–1430 nm provided excellent contrast between the two leaves. Such images displayed two well-resolved modes on the histogram, where each mode corresponded to a leaf with a different moisture level. For example, a 1529/1416 nm image of 2 h and 12 h leaves (Fig. 7a) showed clear visual contrast and high *D*_*B*_ = 5.128. This contrast was significantly better than the single-mode images recorded at 1529 nm (*D*_*B*_ = 0.192) or 1416 nm (*D*_*B*_ = 0.318) (Supplementary Fig. 10). As expected, 1529/1416 nm images of the leaves with the same level of moisture provided negligible contrast with a nearly monomodal histogram distribution and low contrast values (*D*_*B*_ = 0.320) (Fig. 7b).

**Figure 8:**
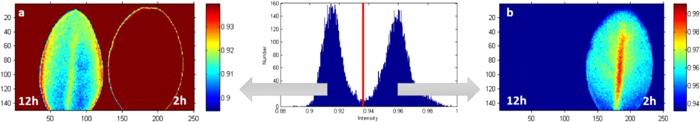
Histogram thresholding of 2 h and 12 h leaves on 1529 nm/1416 nm image. A threshold (red bar) is placed in between the two modes in the histogram, enabling visualization of either a (**a**) dry (left) or (**b**) fresh (right) leaf.

The strong bimodality of 1529/1416 nm enabled efficient thresholding of the image for separating fresh from stressed leaves (Fig. 8). This technique has direct practical application in precision agriculture where plants with lower water content can be identified by selecting an appropriate threshold on the image histogram.

## Comparison of a novel image-derived index 1529/1416 nm with traditional indices.

The developed novel index 1529/1416 nm was compared to several commonly used vegetation indices (i.e. WBI, MSI and NDWI) in the 800–1600 nm spectral range. These indices are broadly used for monitoring the reflectance of vegetation from ground-based remote cameras and from space stations. Although transmission and reflection of the leaves are interrelated^31^ (see also Supplementary Fig. 3 showing a correlation between transmission and reflection spectra in water-stressed boxwood leaves), the comparison provided below should be used with some caution and will require additional validation in the future.

### Water band index (WBI, 900 nm/970 nm)

**Figure 9:**
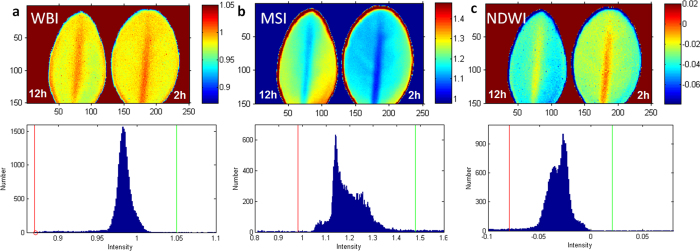
Images and histograms obtained using traditional indices. (**a**) WBI index 900 nm/970 nm. The common range of values for green vegetation is 0.8 to 1.2. (**b**) Moisture Stress Index (MSI) = 1599 nm/819 nm. The common range for green vegetation is 0.4 to 2. (**c**) Normalized Difference Water Index (NDWI) = (857 nm–1241 nm)/(857 nm + 1241 nm). The common range for green vegetation is −0.1 to 0.4. Left leaf −12 h post detachment, right leaf −2 h post detachment. The images correspond to the histogram within specified red and green boundaries and correspond to the scale of intensities.

The WBI is a reflectance measurement that is sensitive to changes in canopy water content^32^,^33^,^34^. The WBI-based image of 2 h and 12 h leaves presented marginal visual contrast between the two (Fig. 9a). Accordingly, the histogram featured almost a monomodal distribution and providing a low contrast value of *D*_*B*_ = 0.181.

### Moisture Stress Index (MSI, 1599 nm/819 nm)

The MSI is another reflectance metric that is sensitive to water content^22^,^35^. The absorption at 819 nm is weakly affected by changing water content, this band is used as a reference. The histogram (Fig. 9b) shows a complex distribution of the overlapped modes with the calculated contrast value of *D*_*B*_ = 0.846, better that WBI, but much lower than that for 1529/1416.

### Normalized Difference Water Index (NDWI, (857 nm–1241 nm)/(857 nm + 1241 nm))

The NDWI is a normalized index also sensitive to changes in vegetation canopy water content^36^,^37^. The histogram of the produced image (Fig. 9c) demonstrates an overlapped bimodal distribution, with a partial separation between the peaks and contrast values *D*_*B*_ = 0.50, significantly lower than that for 1529/1416.

Similar experiments were performed on multiple leaf pairs to yield more comparable results. The results are summarized in Supplementary Table 1. Although some variances were present in the *D*_*B*_ values, the novel index 1529/1416 yielded better results than the traditional indices for all studied leaves. The differences in *D*_*B*_ values can be attributed to the fact that the leaves were collected at different times and the water contents vary due to weather conditions.

## Conclusions and Future Work

The goal of the presented study was to design a practical and versatile small-scale (single plant) platform in SWIR and algorithms to define image-based indices of plants, with potentially better sensitivity than currently used metrics. Imaging in the SWIR range has distinct advantages over imaging in the visible range that lie mainly in the presence of highly abundant endogenous plant chromophores, such as water, allowing plant differentiation based on wavelength-dependent optical properties.

The minimum significant change detected by the spectroscopy method in SWIR (in the reflection mode) was reported to be 52%^22^, leading the authors to conclude that indices derived from this spectral range cannot be applied to detect water stress. Our approach to increase sensitivity was based on a search of image-derived indices, rather than spectroscopy-derived readings, using the HIS-SWIR technique with high spectral resolution. In differentiating moderately water-stressed leaves, we relied on histogram analysis using Bhattacharyya metrics to quantify the contrast.

In this work the search for image-derived indices was performed by two methods, using a ratio of intensities (SRC, Weber and Michelson) and via histogram image analysis. The ratio of intensities metric largely failed to select indices that provide the best visual contrast. We found that the best visual contrast between two leaves correlates with the high level of bimodality on the histogram plot. Stronger separation between the modes provides better contrast. Analysis of the contrast using the histogram approach and Bhattacharyya metrics demonstrated the advantage of image-derived indices over the traditional spectroscopy based readings. With this strategy, we achieved sensitivity to less than 20% in water stressed plants.

The far-reaching goal of this study is to provide an imaging configuration to meet the specific application requirements for monitoring plants in the field. Based on the results of this or similar studies with other type of plants we plan to design a transportable imaging system. The system will utilize a 2D CCD SWIR camera with index-specific filters and aberration corrected lenses. The filter parameters, such as the central wavelength and the bandwidth, will be simulated using the software to achieve the suitable contrast. Based on the simulating studies, the filters (two or more) with the optimized characteristics will be custom fabricated and integrated into the imaging system to use in the field studies.

To further increase the specificity of the instrument, the spectral range has to be expanded from the 800–1600 nm to much broader range 400–2400 nm, the current standard in commercial spectroscopy-based systems^38^. The spectral range of our imaging system is largely limited by the InGaAs sensor. Increasing the range to visible (400–800 nm) can be achieved by an additional Si- based detector or using recently introduced hybrid detectors covering the range of 400–1700 nm. Going beyond 1700 nm is a more challenging task because of the lack of sensitive detectors, as well as efficient light sources operating at longer wavelengths. Recently developed InGaAs sensors are able to shift the cutoff wavelength from 1700 to 2500 nm^39^,^40^. However, these detectors have higher dark current characteristics resulting in poorer device performance, such as a low signal to noise ratio. If developed, such broadband imaging systems will enable the discovery of novel optical features across the full spectral range of plants.

Overall, using naturally desiccated leaves as models and the hyperspectral SWIR imager, we provided a strategy for deriving image-derived indices, enabling highly sensitive analysis of plants. The proposed quantitative aspects of the bimodality can serve as criteria for future development of an automatic non-supervised search for the best contrast. With further refinement of the algorithms and the hardware to larger spectral range, the sensitivity can be additionally improved and expanded to other plant components for early diagnostics of a variety of stressors.

## Methods and Materials

### Leaves preparation

Leaves of American boxwood plants (*Buxus, sempervirens ‘Arborescens’*) were randomly collected from commercially purchased plants grown in St. Louis, MO in full sun. Boxwood leaves retain their green color throughout the year and remain visibly green under prolonged storage conditions, minimizing potential effects caused by the alteration of green color on SWIR images. Boxwood leaves are also the optimal size for fitting on the stage of the imaging platform used in this study. Leaves discussed in this paper were collected in July and November of 2014 and July 2015.

### Relative water content of leaves using weight and integrating sphere method

Leaves of similar size were randomly separated into two groups: controls and samples. The control group was hydrated to full turgidity for 3–4 h in a refrigerator at 8 ^o^C by floating them in a vial with de-ionized water. After hydration, the leaves were dried of any surface moisture with tissue paper prior to weighing them to obtain fully turgid weight (TW). Leaves from the control group were then dried in oven for 24 h under vacuum (25 torr) at 80 ^o^C until the weight of the leaves stabilized. The stabilized weight corresponded to the dry weight (DW). RWC was calculated according to eq. 1^20^. Another group of leaves (samples) was placed in a Petri dish under standard laboratory conditions; leaf weight from this group was recorded at certain time points within a 2–50 h time range.


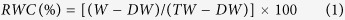


where, *W* – sample weight, *TW* – sample turgid weight, *DW* – sample dry weight.

Reflection spectra of the leaves were recorded using a custom setup based on an integrating sphere of 6” diameter with two ports and a sample tray on the bottom of the sphere connected to a fiber optic bundle with mirrors to reflect the photons to an imaging spectrograph with an InGaAs diode array detector (see reflection and transmission geometries in Supplementary Fig. 1). Both, reflectance and transmittance were recorded. The reflectance and transmittance values were defined by the ratio of reflected radiant power to incident radiant power.


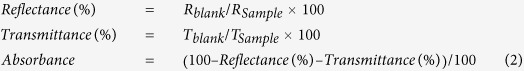


The spectra in the integrating sphere were collected 20 times per measurement and averaged to minimize the noise.

### Imager design

The imager was assembled for raster-scanning in transillumination geometry, as we previously described^26^,^41^ with some modifications (see Supplementary Fig. 6). Leaves were placed on a glass stage and illuminated with a halogen lamp through fiber optics and an objective with relatively high transmission in the NIR, as defined previosly^29^. The integration time of the detector was adjusted to achieve optimal tradeoff between photon counting and scan time. Samples were measured with 100 μm resolution.

### Data analysis

Raw data were analyzed using an in-house developed software package based on MATLAB. Each scanned area of sample generated a data file consisting of light intensity values at wavelengths ranging from 800–1600 nm in 1.56 nm increments (total 512 datapoints for each pixel). The data files were used to create images through a custom graphical user interface (GUI) program. The contrast metrics Simple Ratio Contrast (SRC), Weber Contrast (WC) and Michelson Contrast (MC) were calculated according to the following equations:


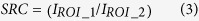



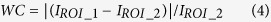



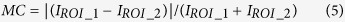


where *IROI*_*1*_ and *IROI*_*2*_ are the intensities of the two region of interests (ROIs).

### Histogram analysis

A bimodal test of the image histogram was performed in MATLAB by fitting a histogram with a two-member Gaussian distribution model (eq. 6) within the boundary conditions specified for each image histogram. Goodness of fit was judged by visual observations, residual plots, and the R-square value. The output provided the heights, means, and widths for each peak:


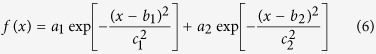


where the parameter *a* is the height of the curve’s peak, *b* is the position of the center of the peak (mean), and *c* is the width of the peak.

Identified fitting parameters, such as means and peak widths (converted into the standard deviation of the peak), were plugged into the Bhattacharyya equations to obtain *D*_*B*_ and *C*_*B*_. (eq. 7)


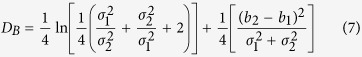


Where the standard deviation *σ* and coefficient *c* are related:


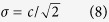


The Bhattacharyya coefficient was calculated according to eq. 9


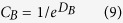


Fitting curves had relatively high goodness of fit *R*^*2*^ > 0.96 (Table 1, Supplementary Fig. 6).

## Additional Information

**How to cite this article**: Kim, D. M. *et al.* Highly sensitive image-derived indices of water-stressed plants using hyperspectral imaging in SWIR and histogram analysis. *Sci. Rep.* **5**, 15919; doi: 10.1038/srep15919 (2015).

## Supplementary Material

Supplementary Information

Supplementary Movie

## Figures and Tables

**Table 1:** Quantitative characterization of common and image-derived indices.

Index	Wavelengths/bands	*D*_*B*_	*C*_*B*_	*R*^*2*^
*Image guided indices (ratiometric)*				
Image-guided index	1529 nm/1416 nm	5.128	0.006	0.970
SRC	1459 nm/1129 nm	1.142	0.319	0.964
Weber (WC)	(1459–1129 nm)/1129 nm	1.142	0.319	0.964
Michelson (MC)	(1460 nm–1421 nm)/(1460 nm + 1421 nm)	0.239	0.788	0.989
*Band indices*				
Narrow band*	From 1300 to 1500 nm	0.777	0.460	0.982
Broadband*	From 800 to 1600 nm	0.286	0.751	0.970
Single band*	1416 nm	0.318	0.728	0.970
Single band*	1529 nm	0.192	0.826	0.985
*Traditional indices (bandratios)*				
WBI	900 nm/970 nm	0.181	0.834	0.998
MSI	1599 nm/819 nm	0.846	0.429	0.972
NDWI	(857 nm–1241 nm)/(857 nm + 1241 nm)	0.500	0.607	0.989

*D*_*B*_ - Bhattacharyya distance, *C*_*B*_ - Bhattacharyya coefficient, *R*^*2*^ *–*goodness of fit of the bimodal Gaussian distribution, *Referenced to light intensity.
