# Supplementary material for: Highly sensitive image-derived indices of water-stressed plants using hyperspectral imaging in SWIR and histogram analysis
Source: Sci Rep. 2015 Nov 4;5:15919. doi: 10.1038/srep15919 (PMC4632122; doi:10.1038/srep15919)
Supplement: Supplementary Information [file srep15919-s1.pdf]

SUPPLEMENTARY INFORMATION
Highly sensitive image-derived indices of water-stressed plants using hyperspectral imaging in SWIR and histogram analysis

David M. Kim[1], Hairong Zhang[1], Haiying Zhou[1], Tommy Du[1], Qian Wu[1], Todd C. Mockler[2], Mikhail Y. Berezin[1*]

[1]Department of Radiology, Washington University School of Medicine, St. Louis, MO 63110, [2]Donald Danforth Plant Science Center, St. Louis, MO 63132

A: Reflection geometry

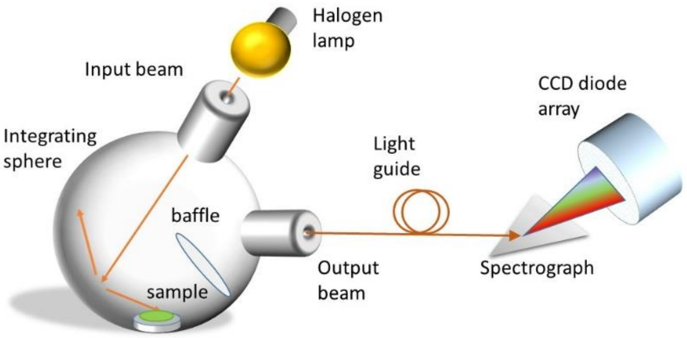

B: Transmission geometry

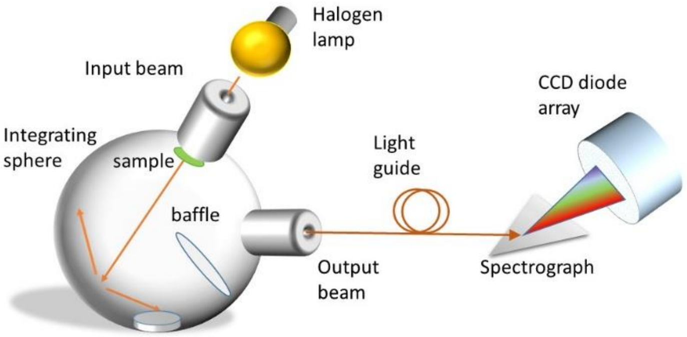

Figure 1: Measurement of leaves optical properties in an integrating sphere setup. A: Reflection geometry. B – Transmission geometry. The spectra of the leaves were recorded using a custom setup based on an integrating sphere 6" diameter with two ports and a sample tray on the bottom of the sphere (Quanta-phi, Horiba), connected to a fiber optic bundle with mirrors to reflect the photons to an iHR 320 imaging spectrograph (Horiba), focal length 320 mm, aperture f/4.1 with 100 grove/mm, blaze 800 grating. An air cooled halogen lamp (20W, HL-2000 HP, Ocean Optics) was used as a light source and attached to the integrating sphere through a port on top of the sphere, in such a way that the light was pointed "out" from the sample for uniform light

distribution. A liquid nitrogen cooled InGaAs diode array CCD camera Symphony (Horiba) was used as a detector in a ‘high sensitivity’ mode. Integration time 0.2 s and slit 2 nm were used for all the experiments. An intact leaf was placed on the bottom of the integrating sphere for reflection geometry, or in front of the entrance port (transmission geometry). The spectra were corrected for light intensity and dark noise and referenced to the spectrum of the integrating sphere. Each spectrum was collected 20 times and averaged.

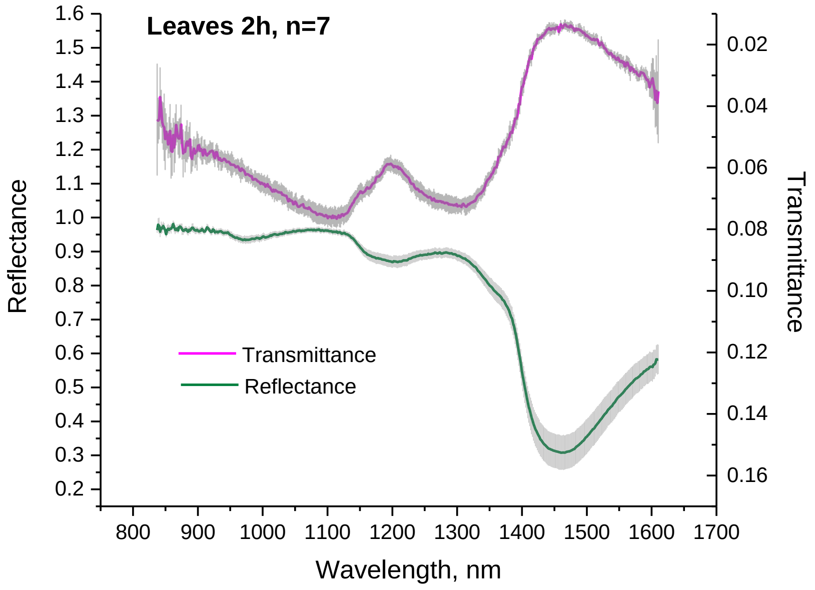

Figure 2: SWIR transmittance and reflectance spectral variability of boxwood leaves randomly sampled from a plant 2h post-detachment, n = 7

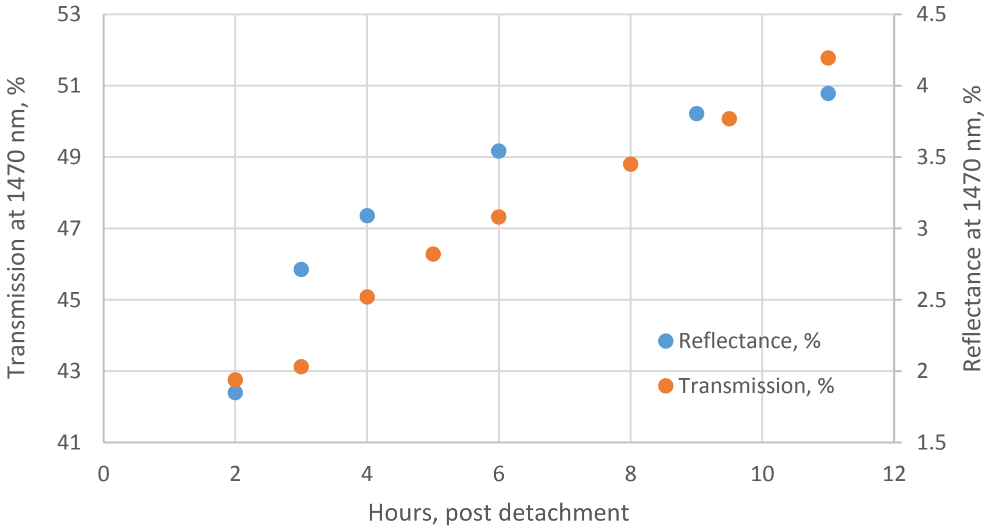

Figure 3: Correlation between the reflection and transmission of leaves at 1470 nm

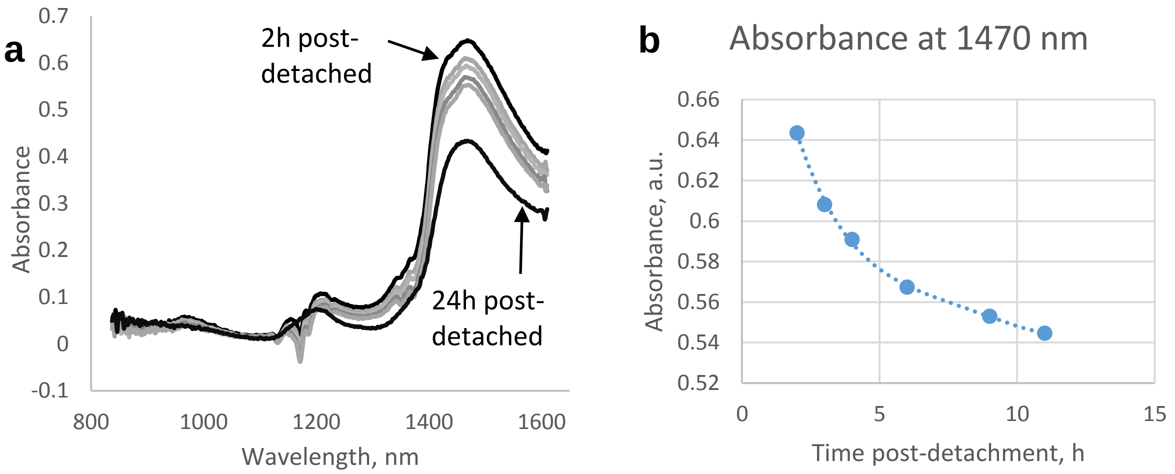

Figure 4: (a) Absorbance spectra calculated from the reflection and transmission data. (b) The change in absorbance spectra at 1470 nm.

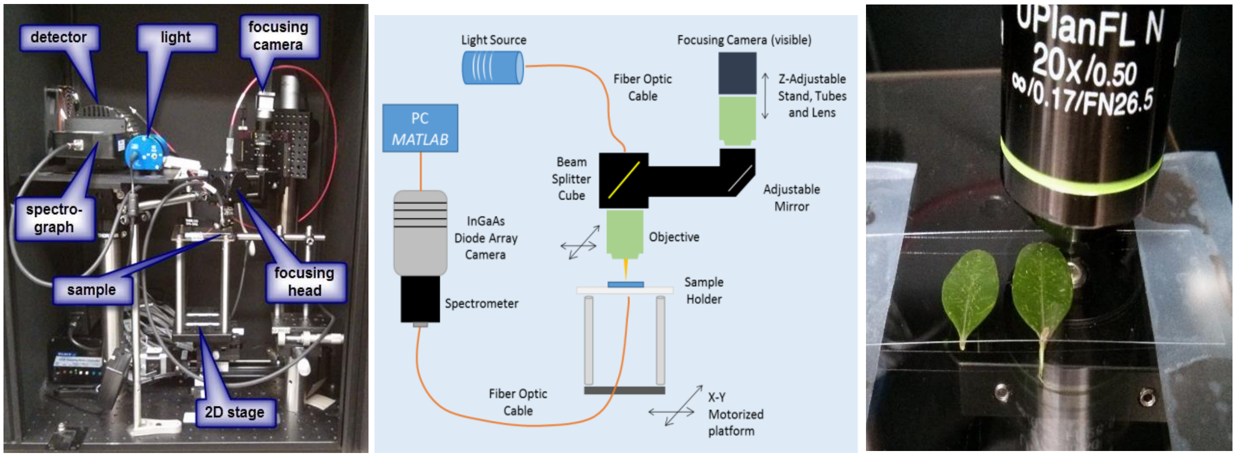

.

Figure 5: **The design of the imager used for data acquisition of a datacube in SWIR**. Left: Raster scanning machine. Middle: Schematic of the imager. Right: close-up photo of the imaging setup). Leaves were placed on a glass stage supported by two orthogonally overlaying XSlide step motors (Velmex Inc.) A halogen lamp (7 W, Ocean Optics, HL-2000) with a stable spectral output in SWIR was coupled through a large-diameter NIR-rated optical fiber (Ø1000 µm, 0.39 NA, Thorlabs) to an objective UPLan FLN 20x/0.50 (Olympus) for focusing the light. This objective has relatively high transmission in NIR compared to other tested objectives[52]. The light through the sample was captured by another optical fiber (Ø400 µm, 0.48 NA, Thorlabs) that was connected to Nunavut CCD detector (Bayspec) operated at "high sensitivity" mode and featured InGaAs 512 pixel linear diode array sensor thermoelectrically cooled to $-50^{o}C$. The signal from each pixel of this light-sensing photodiode element is proportional to the number of incident photons, insuring linear response over a wide range of light intensities across the spectral range, giving such systems quantitative capabilities similar to imaging spectrophotometers. Spectral resolution of ~1.56 nm/pixel was achieved with a spectrograph with volume phase grating (VPG), f/1.8 design with a range of 800 nm to 1600 nm (Bayspec). The integration time of the detector

was adjusted to achieve optimal tradeoff between photon count and scan time. Samples were measured with 100 μm resolution.

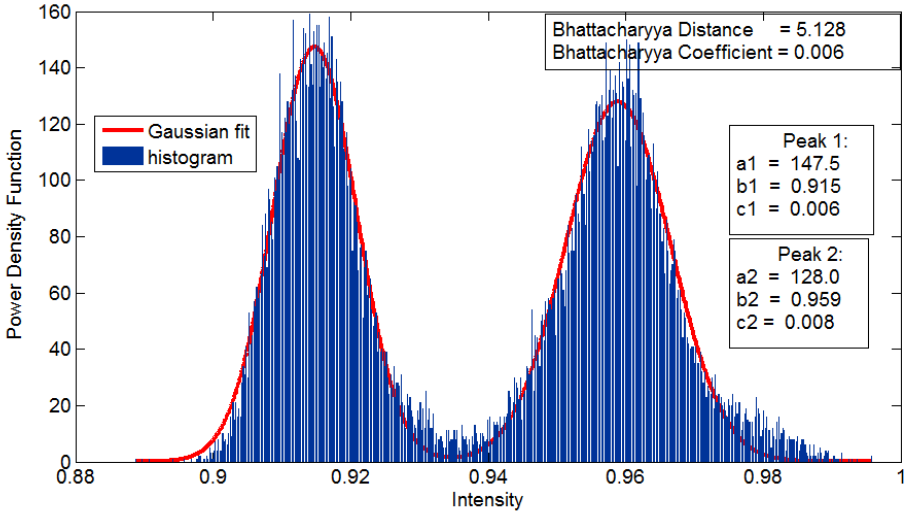

Figure 6: Example of a fit of the image histogram using bimodal Gaussian distribution at 1529 nm/1416 nm. Coefficients: a – relates to the intensity of the peak, b – to the mean of the peak, and c – to the width of the peak

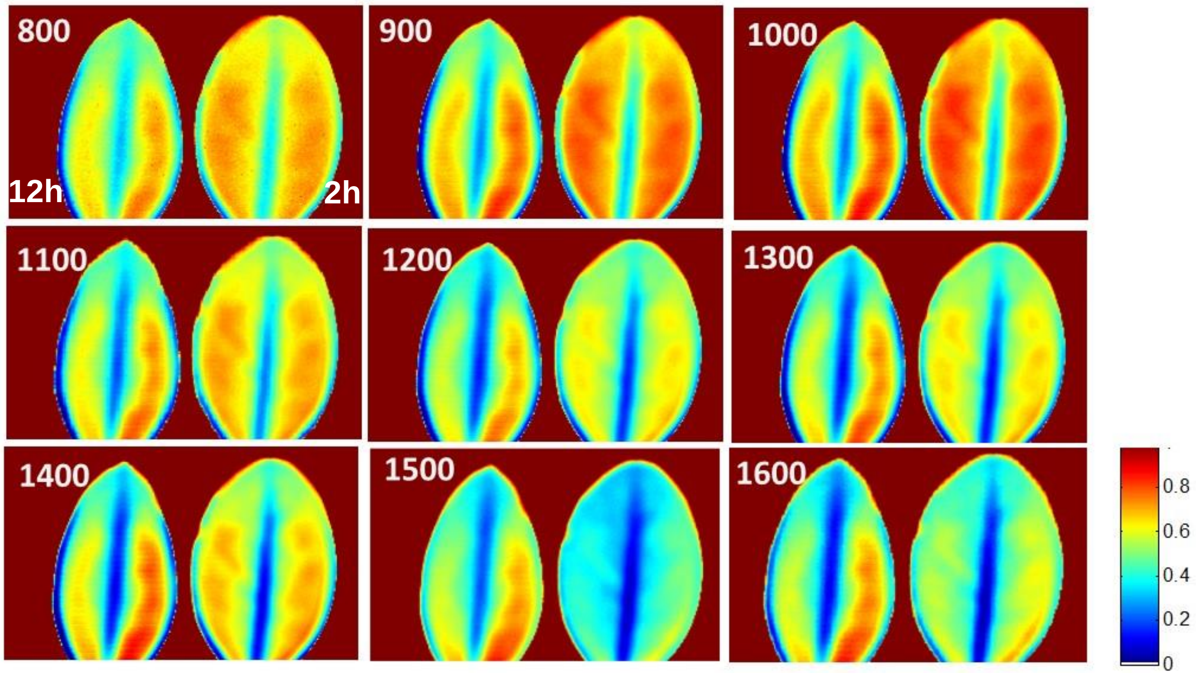

Figure 7: Transmission images of leaves with 20% difference in moisture level at selected wavelengths from 800 to 1600 nm. A leaf on the left has 20% less moisture (12h post-detachment) and shows higher transmission. Pixel intensities were normalized to the intensity of

the light source and the images were optimized for the best contrast by adjusting the corresponding image histogram. Higher intensity on the jet colored image corresponds to higher transmission.

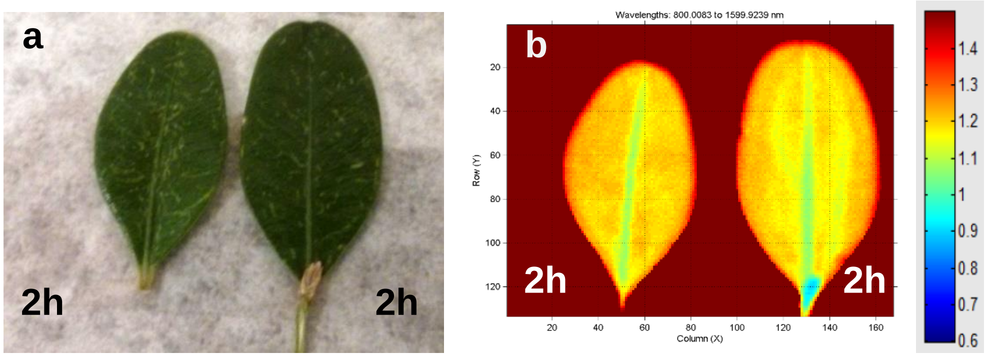

Figure 8: Leaves with the same level of moisture (2h post-detached). Fresh leaves were collected in November 2014. (a) Reflection image with the visible color camera (made with camera Nikon 1J, objective 1 NIKKOR 18.5 mm f/1.8). (b) Broadband transmission image of the leaves using hyperspectral setup 800 – 1600 nm.

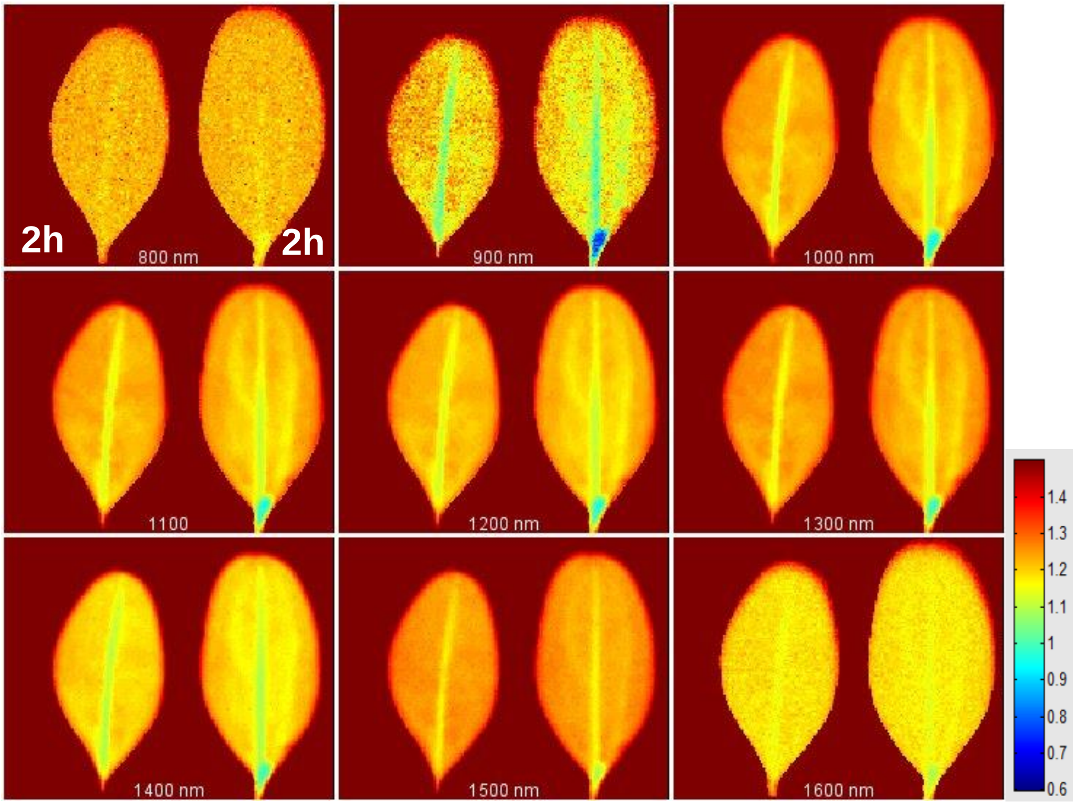

Figure 9: Transmission images of leaves with a similar level of moisture at selected wavelengths from 800 to 1600 nm). Increment 100 nm. Image intensities were normalized to the intensity of the light source. The images were optimized for the best contrast by adjusting the corresponding image histogram. Higher intensity on the jet colored image corresponds to higher transmission.

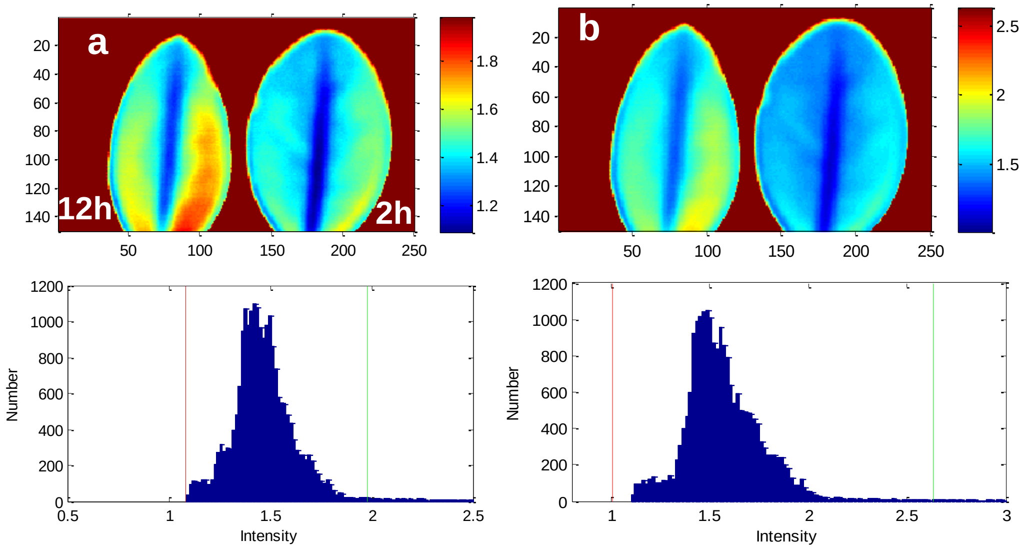

Figure 10: Transmission images of leaves with different level of moisture. Shown 2h and 12h post-detachment with the corresponding histograms (a) Monochromatic image at 1529 nm. (b): Monochromatic image at 1416 nm. All images are shown for pixels within given boundaries on the corresponding histogram.

Table 1: Comparison of indices between different leaf pairs taken at the different time of the year (2h and 12h post detachment)

**Index**		*Image-guided index*	*MSI*
**Wavelengths/bands**		1529 nm / 1416 nm	1599 nm / 819 nm
**D_B**	Leaf pair 1 (used in main text)	5.128	0.846
	Leaf pair 2	11.144	2.238
	Leaf pair 3	1.73	1.017
**C_B**	Leaf pair 1	0.006	0.429
	Leaf pair 2	0	0.107
	Leaf pair 3	0.206	0.362
**R^2**	Leaf pair 1	0.970	0.972
	Leaf pair 2	0.924	0.929
	Leaf pair 3	0.941	0.976

D_B - Bhattacharyya distance, C_B - Bhattacharyya coefficient, R^2 –goodness of fit of the bimodal Gaussian distribution, *Referenced to light intensity. Leaf pair 1: July 24 2013, leaf pair 2: July 27 2015, leaf pair 3: July 28 2015.
